# Translation regulation in the spinal dorsal horn – A key mechanism for development of chronic pain

**DOI:** 10.1016/j.ynpai.2018.03.003

**Published:** 2018-04-03

**Authors:** Shannon N. Tansley, Calvin Wong, Sonali Uttam, Jeffrey S. Mogil, Arkady Khoutorsky

**Affiliations:** a Department of Anesthesia, McGill University, Montréal, QC H3A 0G1, Canada; b Department of Psychology, McGill University, Montréal, QC H3A 1B1, Canada; c Alan Edwards Centre for Research on Pain, McGill University, Montréal, QC H3A 0G1, Canada

## Abstract

Chronic pain is a pathological condition characterized by long-lasting pain after damaged tissue has healed. Chronic pain can be caused and maintained by changes in various components of the pain pathway, including sensory neurons, spinal cord and higher brain centers. Exaggerated sensitivity and responsiveness of spinal nociceptive circuits, representing maladaptive plasticity, play key roles in the amplification of peripheral signals in chronic pain conditions. This spinal amplification mechanism profoundly contributes to the development and maintenance of chronic pain hypersensitivity in response to peripheral injury, and in some cases occurs independently of the peripheral stimulus. Long-lasting changes in the activity of spinal neurons are caused by alterations in their cellular proteome, which relies on *de novo* gene expression. Recent evidence indicates that translational control of gene expression plays a major role in determining protein levels, and is intricately involved in different forms of intrinsic and synaptic plasticity. In this review, we summarize findings supporting a key role for translational control in spinal cord-dependent mechanisms of chronic pain, and present recent approaches to reverse persistent pain by targeting these mechanisms.

## Highlights

• Spinal sensitization shares molecular mechanisms with hippocampal LTP and memory.
• Changes in mRNA translation are observed in many chronic pain conditions.
• Targeting translational control mechanisms is a promising strategy to inhibit pain.
• Targeting spinal reconsolidation can reverse established hypersensitivity.

## Introduction

Peripheral injury causes acute pain, which is essential for an organism’s survival by ensuring quick withdrawal from harmful or potentially harmful stimuli. Under most circumstances, pain resolves shortly after damaged tissue heals. However, in some cases, the pain does not subside and persists after full tissue recovery. This type of pain, called chronic pain, does not serve any protective function and is likely driven by pathological changes that can arise in different components of the pain pathway. Long-lasting sensitization of primary sensory neurons and spinal nociceptive circuits, and plastic changes in brain regions, have all been associated with enhanced transmission and sensation of pain. In this review, we will focus on the spinal cord dorsal horn, which integrates inputs from peripheral and descending pathways to generate an output that is transmitted up to the brain. First, we will briefly describe the mechanisms underlying the sensitization of spinal pain circuits, and then present evidence for the role of translational control in the regulation of these processes.

## Mechanisms underlying sensitization of spinal nociceptive circuits

In chronic pain conditions, repeated or intense noxious stimuli lead to maladaptive plastic changes along the pain pathway, including a sensitization of spinal nociceptive circuits, a phenomenon known as central sensitization (Woolf, 2011). Central sensitization is considered to be a key mechanism underlying the development of persistent hypersensitivity states (Latremoliere and Woolf, 2009). Alterations in several cellular processes can contribute to central sensitization, including enhanced postsynaptic response of spinal neurons to neurotransmitter release from primary afferents (Ikeda et al., 2003, Ikeda et al., 2006), reduced inhibitory tone as a result of decreased excitability of spinal inhibitory interneurons (Guo and Hu, 2014, Torsney and MacDermott, 2006), and inefficient GABAergic and glycinergic neurotransmission (Coull et al., 2003), as well as modulation of descending pathways (Ossipov et al., 2014). An imbalance of excitatory versus inhibitory activity in central sensitization leads to enhanced excitability of spinal nociceptive circuitry, which causes an amplification of the peripheral signal. Central sensitization results in a reduced pain threshold (allodynia), an increase in the perceptual response to noxious stimuli (hyperalgesia), and a recruitment of peripheral inputs from non-injured areas, causing an expansion of the receptive field (secondary hyperalgesia).

## Translational control of neuronal plasticity

Long-lasting modulation of intrinsic excitability and synaptic functions relies on new gene expression. Gene expression can be modulated at different steps: transcription, mRNA translation, mRNA and protein stability, and post-translational modifications of protein. Translational control allows for the modulation of the cellular proteome by regulating the efficiency by which mRNA is translated into proteins. It provides neurons with a mechanism to quickly and locally respond to intracellular stimuli and extracellular cues by modifying their cellular or synaptic proteome.

### Translational control mechanisms

**Fig. 1:**
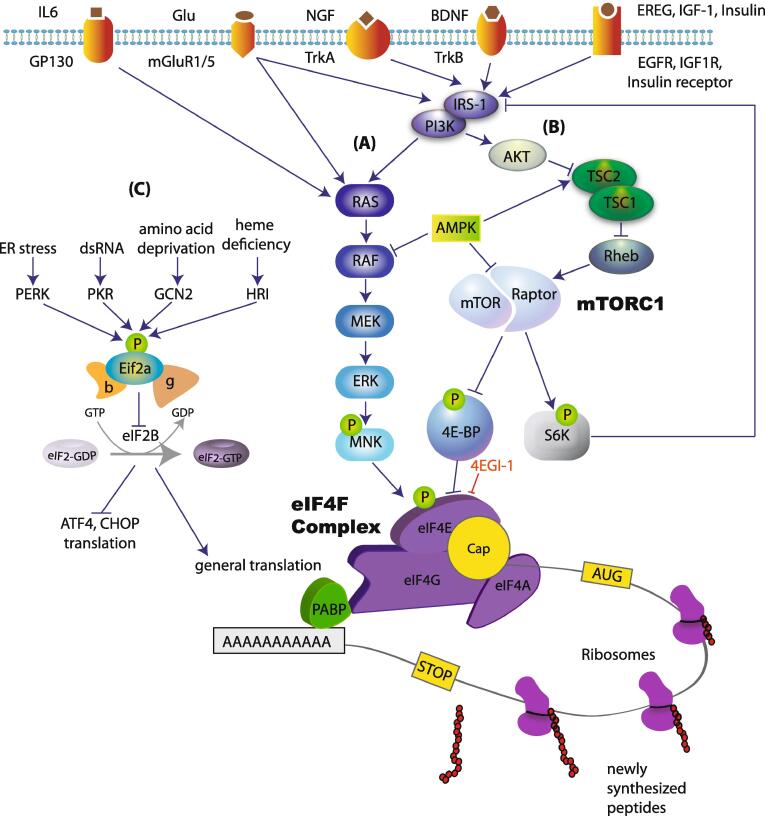
Translational control mechanisms. Signaling pathways upstream of translation can be stimulated by activation of several membrane receptors. The activation of these receptors leads to subsequent stimulation of (A) RAS/RAF/ERK pathway and the phosphorylation of eIF4E, and (B) the activation of PI3K/AKT/mTORC1 pathway. mTORC1 phosphorylates and inhibits the translational repressor 4E-BP, resulting in increased eIF4F complex formation, which promotes the recruitment of the ribosome to the cap structure at the 5′ end of the mRNA. This mechanism controls translation of a specific subset of mRNAs. (C) Translation is also regulated via eIF2ɑ pathway, which controls both general translation and translation of mRNAs containing uORFs at their 5′ UTR (e.g. ATF4 and CHOP).

mRNA translation can be divided into three stages: initiation, elongation and termination. Initiation is the rate limiting step for translation and therefore is tightly regulated by several mechanisms (Sonenberg and Hinnebusch, 2009). At their 5′ end, all nuclear transcribed eukaryotic mRNAs contain a structure called 7 methylguanosine triphosphate (m^7^Gppp), termed the “cap”. This structure facilitates ribosome recruitment to the mRNA (Fig. 1). The 3′ end of the mRNA contains a poly(A) tail that protects mRNA from degradation, and binds poly(A)-binding protein (PABP). The mechanisms regulating translation initiation can be divided into two major categories: (1) regulation of the recruitment of the ribosome to the cap at the 5′ end of mRNA (via phosphorylation of translation initiation factors such as 4E-BPs, eIF4E and eIF2a), and (2) regulation of translation at the 3′ end of mRNA via controlling the length of the poly(A) tail (e.g. by CPEB).

Ribosome recruitment requires a group of translation initiation factors, termed eIF4 (eukaryotic initiation factor 4). A critical member of this group is eIF4F, which is a three-subunit complex (Edery et al., 1983, Grifo et al., 1983) composed of (1) eIF4A (an RNA helicase), (2) eIF4E, which specifically interacts with the cap structure (Sonenberg et al., 1979) and (3) eIF4G, a large scaffolding protein that binds to both eIF4E and eIF4A. eIF4G serves as a modular scaffold that assembles the protein machinery to direct the ribosome to the mRNA (Fig. 1). eIF4E generally exhibits the lowest level of expression of all eukaryotic initiation factors. It plays a central role in cap-recognition, and due to its low levels of expression, it is considered the rate-limiting step for translation, and a major target for regulation. The assembly of eIF4F is promoted by the mechanistic target of rapamycin complex 1 (mTORC1), which phosphorylates and thereby inactivates translational repressors, the eIF4E-binding proteins (4E-BP1, 4E-BP2 and 4E-BP3). 4E-BPs repress the formation of the eIF4F complex by competing with eIF4G for a common binding site on eIF4E. Upon phosphorylation by mTORC1, 4E-BP binding to eIF4E is reduced, allowing eIF4F complex formation and initiation of translation. mTORC1 also phosphorylates its second major downstream effectors, p70 S6 kinases (S6K1/2), which regulate translation initiation (via eIF4B), translation elongation (via eEF2K) and ribosome biogenesis (via ribosomal protein S6).

eIF4E activity is also regulated via phosphorylation at serine 209 by MNK1/2 (mitogen-activated protein kinase (MAPK) interacting protein kinases 1/2) downstream of ERK (extracellular-signal-regulated kinase) (Fig. 1). This phosphorylation event is associated with increased rates of translation initiation (Gkogkas et al., 2014, Scheper et al., 2002), although the exact underlying molecular mechanism remains unknown.

A second major translational control mechanism is mediated by the translation initiation factor, eIF2 (composed of three subunits) (Sonenberg and Hinnebusch, 2009), via phosphorylation of its α subunit (Fig. 1). Translation initiation requires the formation of a ternary complex composed of the initiator (Met-tRNA_i_^Met^) and the GTP-bound eIF2. At the end of each round of ribosome recruitment, there is a recycling of inactive GDP-bound eIF2ɑ to active GTP-bound eIF2 by the guanine nucleotide exchange factor (GEF), eIF2B (Pavitt et al., 1998). Phosphorylation of eIF2ɑ at serine 51 inhibits the activity of eIF2B, reducing ternary complex formation and thereby inhibiting protein synthesis. Paradoxically, eIF2ɑ phosphorylation stimulates translation of mRNAs containing upstream open reading frames (uORFs) in their 5′ UTRs, such as ATF4 and CHOP. eIF2ɑ is phosphorylated in response to different cellular stress conditions via activation of eIF2ɑ kinases (PERK, PKR, GCN2 and HRI) (Trinh and Klann, 2013). Phosphorylation of eIF2ɑ is largely involved in the regulation of general translation, whereas eIF4E-dependent translational control regulates the translation of a distinct subset of mRNAs, many of which are involved in proliferation, growth and synaptic plasticity.

Translation is also regulated via 3′ end-mediated mechanisms. Translation of mRNAs containing the cytoplasmic polyadenylation elements (CPE) at their 3′ UTR is regulated by the cytoplasmic polyadenylation element-binding protein (CPEB) (Richter and Klann, 2009). CPEB binds CPE and stimulates the prolongation of the poly(A) tail by regulating the polyadenylation apparatus composed of poly(A) polymerase Gld2, deadenylase PARN, and translational factor neuroguidin (Ngd) (Ivshina et al., 2014, Udagawa et al., 2012). Elongation of the mRNA poly(A) tail leads to stabilization of the mRNA and enhanced binding of the poly(A)-binding protein (PABP), which facilitates translation initiation by simultaneously binding to both the poly(A) tail and eIF4G, resulting in mRNA circularization (Gray et al., 2000, Kahvejian et al., 2001). This mechanism has been shown to regulate the translation of *CamkII*α and *Nr2a* mRNAs (Huang et al., 2002, Wu et al., 1998).

### Synaptic plasticity

Synaptic plasticity refers to the ability of the synapse to strengthen or weaken in response to experience or stimuli. The predominant cellular model for synaptic plasticity is long-term potentiation (LTP), which is thought to underlie learning and memory (Morris, 2003). Co-activation of pre- and post-synaptic compartments triggers calcium influx into neurons, stimulating several signaling pathways to promote transcription and translation of plasticity-related genes. The newly synthesized mRNAs are either subsequently translated in the cell body or transported to synapses where they are locally translated (Jung et al., 2014, Tom Dieck et al., 2014). The local protein synthesis model is consistent with the presence of translation machinery (ribosomes and translation factors) and mRNAs in, or close to dendritic spines (Steward and Fass, 1983, Steward and Levy, 1982). Moreover, LTP-inducing stimulation causes ribosomes to move from dendritic shafts to spines with enlarged synapses (Ostroff et al., 2002). Protein synthesis in dendrites occurs in response to various forms of stimulation (Kang and Schuman, 1996, Scheetz et al., 2000) and is essential for long-term plasticity (Huber et al., 2000, Kang and Schuman, 1996). Accordingly, studies in the hippocampus, amygdala and cortex have demonstrated a key role of translational control in the protein synthesis-dependent late phase of long-term potentiation (L-LTP), long-term depression (LTD) and learning and memory (Costa-Mattioli et al., 2009). Inhibition of translation with anisomycin or inhibitors of mTORC1 impairs L-LTP and long-term memory (LTM) (Cammalleri et al., 2003, Tang et al., 2002). Neuronal activity and behavioural training lead to a reduction in eIF2ɑ phosphorylation, resulting in suppression of LTD and stimulation of L-LTP and long-term memory (Costa-Mattioli et al., 2005, Costa-Mattioli et al., 2007, Costa-Mattioli and Sonenberg, 2006, Di Prisco et al., 2014). Regulation of translation via CPEB and PABP has been also shown to control L-LTP and LTM (Alarcon et al., 2004, Khoutorsky et al., 2013, Richter, 2007, Udagawa et al., 2012).

Most of the current knowledge on the role of translational control in neuroplasticity has been derived from experiments in the hippocampus, however recent studies show that similar mechanisms regulate activity-dependent long-term modification of synaptic strength in other brain areas including cortex, amygdala, and spinal cord (Belelovsky et al., 2005, Buffington et al., 2014, Khoutorsky et al., 2015, Khoutorsky and Price, 2017, Melemedjian and Khoutorsky, 2015, Parsons et al., 2006).

## Translational control in spinal plasticity

Studies of spinal LTP and central sensitization have demonstrated a significant overlap with underlying mechanisms known in hippocampal LTP and memory formation (Ji et al., 2003, Price and Inyang, 2015). LTP of extracellular field potentials in the superficial dorsal horn of the spinal cord can be induced by electrical stimulation of afferent C fibers (Liu and Sandkuhler, 1995), noxious stimulation of peripheral tissue, and nerve damage (Sandkuhler and Liu, 1998, Zhang et al., 2004). Stimulation of the sciatic nerve with the LTP-inducing protocol produced long‐lasting allodynia and thermal hyperalgesia (Ying et al., 2006, Zhang et al., 2005), suggesting that spinal LTP might be a cellular model of injury-induced hyperalgesia (Sandkuhler, 2007). A unique feature of spinal LTP is that it exhibits activity-dependent potentiation of both activated synapses, causing homosynaptic potentiation, as well as non-activated synapses, leading to heterosynaptic potentiation (Kronschlager et al., 2016, Latremoliere and Woolf, 2009). Heterosynaptic potentiation, which is not present in the cortex or hippocampus, is the major form of synaptic plasticity in the spinal cord. Heterosynaptic LTP is a key mechanism for the development of distinct forms of activity-dependent central sensitization manifested by a response to low threshold afferents (allodynia) and spread of pain sensitivity to non-injured areas (secondary hyperalgesia) (Latremoliere and Woolf, 2009).

Inhibition of protein synthesis with either cyclohexamide or anisomycin blocked the late-phase of spinal LTP elicited by C-fiber stimulation but did not affect the induction (early) phase (Hu et al., 2003). Thus, similar to hippocampal LTP, spinal LTP exhibits two distinct phases, an early phase that is protein synthesis independent, and a late-phase that is protein synthesis-dependent (Bliss and Collingridge, 1993). Moreover, *Eif4ebp1^−/−^* mice lacking the translational repressor 4E-BP1 show a reduced threshold for the induction of spinal LTP as well as an increased extent of potentiation (Khoutorsky et al., 2015). These results indicate that spinal LTP exhibits bidirectional dependence on protein synthesis, and suggest that stimulation of mRNA translation in spinal neurons might facilitate the sensitization of spinal nociceptive circuitry and accompanied hypersensitivity in chronic pain conditions.

## Evidence for a central role of translational control in chronic pain conditions

Numerous studies have documented increased activity in signaling pathways upstream of mRNA translation in spinal neurons following acute noxious peripheral stimulation and also in chronic pain conditions. Intraplantar capsaicin (Geranton et al., 2009) or carrageenan (Norsted Gregory et al., 2010) injection increases the number of phosphorylated-S6 (p-S6)-positive neurons in the spinal cord. Likewise, the phosphorylation of mTOR and its two major downstream effectors, S6 and 4E-BP1, are enhanced in the dorsal horn of the spinal cord following acute peripheral inflammation induced with carrageenan (Norsted Gregory et al., 2010) and formalin (Xu et al., 2011). mTORC1 signaling also increases in the dorsal horn of the spinal cord in models of chronic pain including chronic inflammation-induced pain caused by complete Freund’s adjuvant (CFA) (Liang et al., 2013), bone cancer-induced pain (Shih et al., 2012) and nerve injury (Zhang et al., 2013). Consistent with the activation of mTORC1, the signaling of upstream kinases such as PI3K and AKT is also upregulated in these conditions in the dorsal horn of the spinal cord (Pezet et al., 2008, Xu et al., 2011).

The functional role of the stimulation of protein synthesis in spinal neurons following peripheral injury has been extensively studied using various pharmacological approaches. Subcutaneous injection of formalin elicits a biphasic pain response. The early phase pain behaviour (0–10 min) is mediated by activation of nociceptors, whereas the second phase (10–50 min) is thought to result from sensitization of spinal pain circuits. Intrathecal administration of the protein synthesis inhibitor anisomycin, or mTORC1 inhibitor rapamycin, profoundly reduces nocifensive behaviour in the second phase of the formalin test but not the first phase (Asante et al., 2009, Kim et al., 1998, Price et al., 2007, Xu et al., 2011). Consistent with the behavioural effects, formalin-induced hyperexcitability in wide dynamic range dorsal horn spinal neurons is inhibited by rapamycin (Asante et al., 2009). Additionally, intrathecal rapamycin alleviates capsaicin-induced secondary mechanical hyperalgesia, which is caused by sensitization of the spinal cord neurons to the input from capsaicin-insensitive Aδ nociceptors (Geranton et al., 2009).

Inhibition of mTORC1 also efficiently alleviates hypersensitivity in chronic models of pain including chronic inflammation-induced pain (Liang et al., 2013, Norsted Gregory et al., 2010), bone cancer-induced pain (Shih et al., 2012) and neuropathic pain (Asante et al., 2010, Cui et al., 2014, Zhang et al., 2013). Pharmacological evidence for the central role of protein synthesis and its master regulator mTORC1 in the spinal cord in the regulation of hypersensitivity is supported by genetic manipulations of different components of the mTORC1 pathway. For example, mechanical hypersensitivity can be caused by activation of the mTORC1 pathway via spinal deletion of TSC2 (Xu et al., 2014), an upstream repressor of mTORC1, or by spinal ablation of 4E-BP1, a repressor of eIF4F complex formation and cap-dependent translation (Khoutorsky et al., 2015). All together, these studies indicate that mTORC1 activity and protein synthesis are upregulated in the dorsal horn of the spinal cord in multiple acute and chronic pain conditions, and their inhibition efficiently alleviates nociceptive behaviour and pain hypersensitivity.

Another important phenomena in which translational control in the spinal cord plays a central role is “hyperalgesic priming” (Reichling and Levine, 2009). Peripheral tissue injury, causing a transient hypersensitivity, leads to persistent sensitization or “priming” of the nociceptive pathway to subsequent insults (Reichling and Levine, 2009). This form of plasticity persists for many weeks and models a clinical situation of increased risk to develop chronic pain in patients with recurrent tissue injuries. The induction of hyperalgesic priming is mediated via brain-derived neurotrophic factor (BDNF)-dependent activation of the mTORC1 and eIF4F complex formation in the spinal cord, which stimulate the synthesis of PKCλ and PKMζ (Asiedu et al., 2011, Melemedjian et al., 2013). Interestingly, spinal LTP is enhanced in primed animals (Chen et al., 2018), supporting the role of synaptic plasticity in this process. Notably, PKCλ and PKMζ play key roles in the expression and maintenance of hippocampal LTP and memory storage, further demonstrating the similarity between molecular mechanisms underlying persistent pain and memory.

## Translational control in opioid-induced tolerance and hyperalgesia

Sensitization of spinal circuits can be caused not only by peripheral tissue damage and subsequent activation of C fibers, but also by aberrant spinal plasticity in response to drugs. Opioid-induced tolerance and hyperalgesia are two examples of such plasticity, which is commonly observed in both animal models and human patients (Sjogren et al., 1993). Opioid-induced hyperalgesia is caused by chronic opioid administration, which can paradoxically lead to central sensitization and pain (Kim et al., 2014, Lee et al., 2013). Although the etiology of opioid-induced hyperalgesia is poorly understood, there are several proposed mechanisms, including the activation of NMDA receptors and protein kinase C (PKC), upregulation of spinal dynorphins, and stimulation of descending facilitatory pathways (Lee et al., 2011). Opioid-induced tolerance occurs during long-term opioid treatment, requiring escalating doses of opioids to obtain the consistent levels of analgesic effect (Chu et al., 2006). The mechanisms underlying opioid-induced tolerance involve opioid receptor desensitization and down-regulation (Allouche et al., 2014, Williams et al., 2013).

Repeated intrathecal administration of morphine is sufficient to cause tolerance and hyperalgesia, suggesting that spinal cord plasticity plays a central role in these phenomena. Interestingly, a selective μ-opioid agonist, DAMGO, stimulates the AKT/mTORC1 axis and its downstream effectors 4E-BP1 and p70 S6 in non-neuronal cell lines stably expressing the μ-opioid receptor (Polakiewicz et al., 1998). This *in vitro* finding was confirmed in an *in vivo* mouse study showing that repeated intrathecal morphine injections strongly induce mTORC1 signaling, and increase eIF4F complex formation and mRNA translation via activation of the μ-opioid receptor (Xu et al., 2014). Remarkably, inhibition of mTORC1 with rapamycin not only alleviated the development of the morphine-induced tolerance and hyperalgesia, but also reversed the fully established tolerance and hyperalgesia after 6 days of daily morphine administration. The mechanisms by which mTORC1 inhibition decrease the opioid-induced tolerance and hyperalgesia remain unknown. It is tempting to speculate that morphine-induced maladaptive spinal plasticity requires mTORC1 and protein synthesis for its induction and maintenance. Consistent with this hypothesis, mTORC1 inhibition attenuated the upregulation of dorsal horn PKCγ, neuronal nitric oxide synthase (nNOS), and CamKIIα, three key molecules involved in spinal plasticity as well as morphine-induced tolerance and hyperalgesia (Xu et al., 2014). A recent study suggested that opioid-induced tolerance and hyperalgesia require the activity of μ-opioid receptors in nociceptors (Corder et al., 2017). Intrathecal injections are known to target both dorsal root ganglia (DRG) and the spinal cord. Since intrathecal administration of mTORC1 inhibitors can block mTORC1 activity in the DRG, an alternative approach should be used with a selective inhibitor of the mTORC1 in the spinal cord but not in the DRG, for example by spinal intra-parenchymal viral injection to downregulate mTORC1.

## New approaches to reverse the established sensitization by targeting spinal reconsolidation

New gene expression is required for the induction phase of spinal sensitization, but not for its maintenance. As soon as sensitization is established, it is no longer sensitive to the inhibition of protein synthesis (Asiedu et al., 2011, Melemedjian et al., 2013). Likewise, memory formation is sensitive to protein synthesis inhibitors at the acquisition stage, but once the memories are formed, they are consolidated into a stable and protein synthesis-independent trace. The consolidated memories can be retrieved by exposure to a conditional stimulus, rendering it to a labile state that requires protein synthesis and mTORC1 activity for further reconsolidation (Lee et al., 2017, Nader et al., 2000). The fragile nature of the memory trace after the retrieval provides an opportunity to erase it by pharmacological targeting of protein synthesis and mTORC1. The central role of mTORC1 in reconsolidation has been demonstrated for memories associated with electrical foot shock and addictive substances (Barak et al., 2013, Blundell et al., 2008, Stoica et al., 2011), raising the possibility that inhibition of mTORC1 is a potential approach to erase the memory of an adverse event, such as in posttraumatic stress disorder (PTSD). The phenomenon of reconsolidation has been also demonstrated in the spinal cord (Bonin and De Koninck, 2014, Bonin and De Koninck, 2015). Intraplantar capsaicin-induced sensitization was insensitive to protein synthesis inhibition when it was fully established, but became anisomycin-sensitive following reactivation of spinal pain pathways with the second capsaicin administration. Transformation of the established capsaicin-induced sensitization into a labile state requires the activation of second-order spinal neurons and the activity of CaMKIIa and ERK, two molecules involved in spinal synaptic plasticity.

The central role of synaptic plasticity in the reconsolidation phenomenon is further supported by LTP experiments (Bonin and De Koninck, 2014). The fully established spinal LTP could be reversed when the second tetanic stimulation was delivered in the presence of anisomycin. All together, these results show that the established hyperalgesia and spinal LTP could be rendered labile by reactivation of pain circuits, further demonstrating an intimate link between persistent pain and the LTP of spinal nociceptive circuits (Ji et al., 2003). Spinal pain reconsolidation-like effects have also been demonstrated in a model of hyperalgesic priming. The activation of dopamine D_1_/D_5_ receptors coupled with anisomycin reversed persistent sensitization in primed animals (Kim et al., 2015).

The results of pain reconsolidation studies provide a potential novel therapeutic avenue to abolish established sensitization of nociceptive circuits in the spinal cord in chronic pain states. Reactivating the pain memory trace and transforming it into the labile state might allow for erasure of persistent pain via blocking reconsolidation using protein-synthesis inhibitors. Notably, the re-opening of the reconsolidation window by spinal application of AMPA and NMDA could be used for a variety of chronic pain states, where the sensitisation-inducing stimulus is unknown or may no longer be relevant.

## Conclusions

Maladaptive plasticity in the spinal cord is a key mechanism for sensitization of pain circuits and the subsequent development of pain. A central role of translational control in the regulation of synaptic and intrinsic neuronal plasticity in the spinal cord provides an opportunity to target translation control mechanisms to reverse the sensitization state. Recently discovered approaches to open the reconsolidation window by peripheral or central reactivation of nociceptive circuits or activation of dopaminergic pathways provide a promising therapeutic avenue. To fully understand the role and mechanisms of action of translation control in pathological pain states, it is essential to identify the subsets of differentially translated mRNAs in different cell types, pain states and phases of spinal cord sensitization. This information would provide an invaluable resource for better understanding the molecular mechanisms underlying the sensitization of spinal pain circuits and chronification of pain.

## Conflict of interest

The authors declare that they have no conflicts of interest.
